# 3D bioprinting of microorganisms: principles and applications

**DOI:** 10.1007/s00449-023-02965-3

**Published:** 2024-01-31

**Authors:** Josha Herzog, Lea Franke, Yingyao Lai, Pablo Gomez Rossi, Janina Sachtleben, Dirk Weuster-Botz

**Affiliations:** 1 Department of Energy and Process Engineering, TUM School of Engineering and Design, Chair of Biochemical Engineering, Technical University of Munich, Boltzmannstraße 15, 85748 Garching, Germany; 2 TUM Campus Straubing for Biotechnology and Sustainability, Technical University of Munich, Petersgasse 5, 94315 Straubing, Germany

**Keywords:** 3D bioprinting, Hydrogel scaffolds, Bioink, Crosslinking, Microorganisms, Bioprocess

## Abstract

In recent years, the ability to create intricate, live tissues and organs has been made possible thanks to three-dimensional (3D) bioprinting. Although tissue engineering has received a lot of attention, there is growing interest in the use of 3D bioprinting for microorganisms. Microorganisms like bacteria, fungi, and algae, are essential to many industrial bioprocesses, such as bioremediation as well as the manufacture of chemicals, biomaterials, and pharmaceuticals. This review covers current developments in 3D bioprinting methods for microorganisms. We go over the bioink compositions designed to promote microbial viability and growth, taking into account factors like nutrient delivery, oxygen supply, and waste elimination. Additionally, we investigate the most important bioprinting techniques, including extrusion-based, inkjet, and laser-assisted approaches, as well as their suitability with various kinds of microorganisms. We also investigate the possible applications of 3D bioprinted microbes. These range from constructing synthetic microbial consortia for improved metabolic pathway combinations to designing spatially patterned microbial communities for enhanced bioremediation and bioprocessing. We also look at the potential for 3D bioprinting to advance microbial research, including the creation of defined microenvironments to observe microbial behavior. In conclusion, the 3D bioprinting of microorganisms marks a paradigm leap in microbial bioprocess engineering and has the potential to transform many application areas. The ability to design the spatial arrangement of various microorganisms in functional structures offers unprecedented possibilities and ultimately will drive innovation.

## Introduction

3D bioprinting emerged from 3D printing as its own research area, by combining biological manufacturing, additive manufacturing and other fields [1, 2]. Applications for mammalian cells include regenerative medicine, such as engineering of organs and tissues, drug discovery and drug development, and disease modelling, as well as bio-hybrid robotics [2–4]. Next to the continuously increasing relevance of bioprinting of mammalian cells, there is another topic that is currently gaining more and more relevance: bioprinting of microorganisms [5, 6].

More recently, 3D bioprinting has also been used to produce functional materials in which microorganisms are cultured. The advancement and integration of bioprinting techniques specifically for the printing of microorganisms offers the potential for a completely new generation of biologically produced functional materials [7–9]. Utilizing and further developing 3D bioprinting to create functional bacteria-laden structures can help to solve various challenges in diverse application fields, such as therapeutic devices, environmental engineering, and industrial biomanufacturing [10].

Furthermore, bacterial bioprinting exhibits several advantages over traditional 3D bioprinting methods applied to mammalian cells. It is more adaptable and compatible with various printing technologies due to the unique characteristics of bacteria [6]: Bacteria have cell walls and can, for example, by forming spores, withstand adverse conditions such as high temperature, freezing, oxidation, high pressure, X-rays, and UV-rays [8]. Moreover, bacteria’s ability to grow and reproduce rapidly lowers the process requirements for bacterial bioprinting [6]. In terms of printing parameters, bacterial bioprinting allows for a wider range of printing temperatures and speeds while maintaining printing resolution. For instance, using bacterial spores as the active ingredient enables molten deposition printing technology with a temperature of up to 75 °C [7]. Additionally, researchers have explored the use of freeze-dried microbial cells for bioprinting and discovered that they exhibit unique shear thinning characteristics with high cell loads, leading to the development of novel living material systems with enhanced catalytic activity and long-term viability [11]. This overcomes the limitation of low cell load in extrusion bioprinting.

The advantages, variety of different applications and multitude of possible microorganisms to be used, make 3D bioprinting of microorganisms an increasingly studied research field. Therefore, within this review, we summarize current bioprinting techniques and bioinks for microbial bioprinting, including the different ways of crosslinking polymeric networks, that are the basis for 3D bioprinted constructs. Bioinks are specialized biomaterials used in 3D bioprinting, composed of biocompatible polymers and engineered to create precise structures. Furthermore, the current state of research is compiled concerning applications and microorganisms printed so far.

## Bioprinting techniques

It is important to note that different printing processes have varying effects on microorganisms, so it remains necessary to consider specific advantages and disadvantages when selecting a printing method [6]. Therefore, the most important printing techniques are reviewed in detail in the following.

**Fig. 1:**
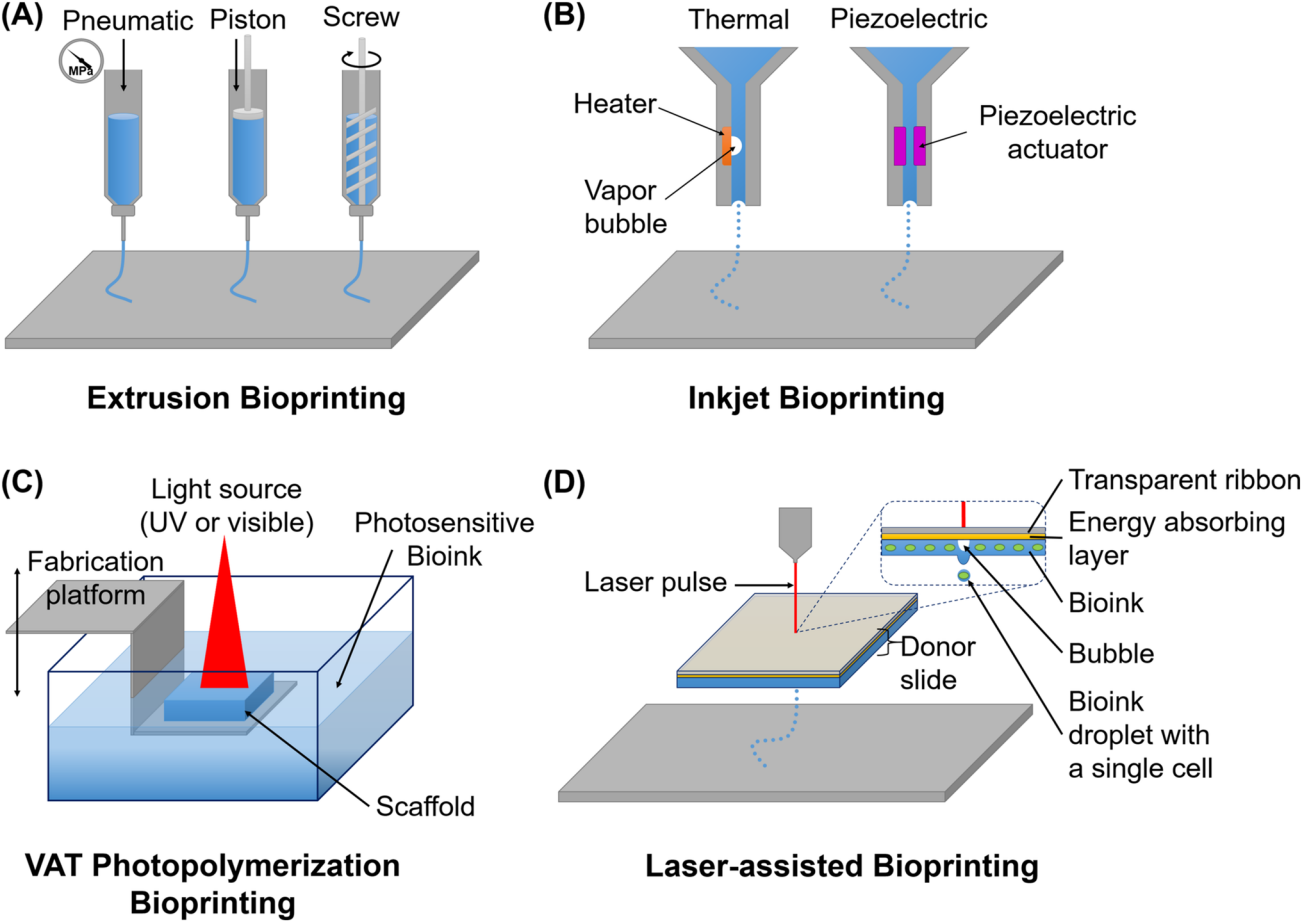
Illustration of the commonly used bioprinting techniques extrusion (**A**), inkjet (**B**), VAT photopolymerization (**C**), and laser-assisted bioprinting (**D**). While, according to the ASTM classification for standards in additive manufacturing, the methods of inkjet and laser-assisted bioprinting are grouped together under the term jetting-based bioprinting, for illustrative purposes, both techniques are considered separately here. **A** Bioink is extruded using pneumatic or mechanical pressure. **B** Thermal inkjet uses heat-induced bubble that pushes the bioink through the nozzle. Piezoelectric actuator produces acoustic waves that propel the bioink through the nozzle. **C** Photopolymerization occurs on the bioinks surface, where light-sensitive bioink is exposed to light energy. **D** Bubble formed by laser pulse propels droplet of bioink. Figure illustration on the basis of Kačarević et al. [13] and Liu et al. [119]

Based on their underlying principle, the printing technologies used in 3D bioprinting can be categorized into four groups: material extrusion (pneumatic, piston and screw driven), inkjet bioprinting, laser-assisted bioprinting and stereolithographic bioprinting [5, 8, 12]. The most commonly used techniques are depicted in Fig. 1 and explained in detail in this chapter.

### Extrusion bioprinting

While micro-extrusion is commonly used in non-biological 3D printing, it serves as a foundation for the extrusion-based approach used in bioprinting. The principles of controlled deposition and layer-by-layer fabrication in micro-extrusion can be adapted to the specific requirements of bioprinting to precisely place bioink. Mechanical [14] or pneumatic [15] pressure extrudes the bioink through a nozzle onto a platform, either or both of which can be moved along x-, y- and z-axis.

An important advantage of extrusion bioprinting is that bioinks can be used that are high in cell density and viscosity [16]. It is also affordable and versatile as commercial fused deposition modeling 3D printers can be modified into bioprinters and therefore customized regarding the specific bioink or structure [17].

Apart from its advantages, extrusion-based bioprinting also has disadvantages. The most important limitation is the low strand resolution of more than 100 μm [18]. Strand resolution refers to the thinnest, but still consistent, filament that extrusion of a particular bioink through the nozzle can provide [19]. This is a problem if certain applications require higher precision when placing the bioink and therefore, in such cases other additive manufacturing techniques with higher resolution are used such as laser-assisted bioprinting [12, 20].

Additionally, extrusion exposes the cells to high shear forces at the nozzle, which can cause mechanical damage. Therefore, numerous bioinks are specifically designed to exhibit shear-thinning behavior [21, 22]. This means that when subjected to an applied shear rate, the apparent viscosity of the bioink decreases. As a result, less pressure is required to extrude the bioink during the printing process, and the shear stress experienced by the bioink is also reduced [23]. The maximum wall shear stress is at its lowest within cylindrical nozzles, compared to tapered conical and conical nozzles, but, because of the longer persistence of this stress alongside the cylindrical nozzle, and because of a lower mass flow rate at the same diameter and inlet pressure, the cell viability is reduced [24]. The versatility in extrusion-based bioprinting also leads to a large range in terms of cell viability (40–80%), which overall is worse than those of other printing principles [15].

### Inkjet bioprinting

The first printers used for bioprinting were adapted versions of commercially available 2D inkjet-based printers [2]. In these modifications, the ink in the cartridge was replaced with biological material, and the paper was replaced with substrates or scaffolds on electronically controlled elevator stages [25, 26]. Nowadays, there are custom-designed inkjet-based bioprinters available, which are optimized for bioprinting, exhibiting increasing resolution, precision, and speed [27]. Inkjet bioprinters are also called drop-on-demand printers because they use thermal or acoustic forces to eject drops of bioink onto a substrate [2].

Thermal-based inkjet bioprinters use heaters, which create bubbles and thus increase pressure in the printhead, ultimately forcing droplets out. During the development of this technique for bioprinting, it was of concern that the thermal element, which reaches temperatures of 200–300 °C, does not damage the cells [28, 29]. This was addressed by several studies which show no impact on the stability of biological molecules such as DNA, as well as an overall temperature increase of only 4–10 °C in the printer head because of the brief heating time of around 2 μs [30, 31]. Several studies have shown that cell viability after printing is about 90% when using thermal inkjet bioprinting with reasonable configurations [29–32], while controlling of the droplet impact velocity and droplet volume in this approach is critical for the viability and proliferation of printed cells [33]. Additionally, higher bio-ink viscoelasticity stabilizes filaments, facilitates precise deposition, improves cell viability, and sustains proliferation by providing added protection to cells within printed droplets even at higher impact velocities [34].

Printers with piezoelectric actuators use rapidly induced changes in shape after applying voltage to eject the bioink [35]. Other inkjet printers use an acoustic radiation force to discharge liquid droplets from an air–liquid interface. This mechanism hinders the use of highly concentrated bioinks, as their viscosity interferes with the ability of acoustic waves to eject droplets smoothly during the printing process [36].

The main disadvantage of both, thermal and piezoelectric-based material jet technologies, is that they are susceptible to frequent nozzle clogging when dealing with highly viscous bioinks as the diameter of the nozzle can be as small as 18 μm [37]. The high viscosity and low concentration of the bioink can hinder the even distribution and deposition of bacteria, resulting in uneven patterns [29].

Inkjet-based bioprinting produces droplets less than 50 μm in diameter. Regarding resolution, it therefore ranks between the less precise extrusion bioprinting and the most precise laser-assisted bioprinting [38].

### VAT photopolymerization bioprinting

Stereolithography (SLA), the additive manufacturing technique that uses VAT Photopolymerization, utilizes either ultraviolet or visible light to solidify photosensitive polymers. In bioprinting, this approach can be adapted by employing photosensitive bioinks. As depicted in Fig. 1C, a laser is used to selectively harden a small amount of bioink. The scaffold forms on a platform that is moved away from the laser afterwards, allowing fresh bioink to flow and coat the structure. This is repeated until a solid 3D structure is formed, and any remaining liquid bioink can be washed away [39, 40].

This technique eliminates the issues caused by shear stress through high pressure in nozzle-based techniques such as extrusion and inkjet bioprinting [41]. Stereolithographic bioprinting enables fast and precise fabrication. Especially detailed structures can be fabricated with resolutions as high as 5 μm [42] and as low as 300 μm [43]. In terms of cell viability, this method can, with a mean microbial viability of 85%, keep up with inkjet bioprinting [41]. The cell viability within this printing technique is mostly influenced by light intensity, wavelength, and photo-initiator concentration [42].

Stereolithographic techniques have become increasingly available in laboratory settings [44] even though they were not usually applied in the context of microbial bioprinting. The group of Dubbin et al. [5] expanded previous SLA techniques and applied them for the first time to microbial bioprinting, as they report a new bioprinting technique to pattern microbial constructs: stereolithographic apparatus for microbial bioprinting (SLAM Bioprinting). With SLAM they were capable of rapidly patterning engineered biofilms with areas of > 48 mm^2^, micrometer-scale X–Y resolution, and thicknesses ranging from 10 μm to > 5 mm [5]. This represents an advantage of stereolithographic bioprinting, as larger surface areas can be crosslinked at the same time, compared to other printing technique.

### Laser-assisted bioprinting

Laser-assisted bioprinting (LAB) is rapidly progressing with microbial cells and holds great promise in addressing various challenges in microbiology and biotechnology [12]. It is also found in literature under the name laser-induced forward transfer (LIFT), which is its underlying principle [20, 45]. LIFT was presented over 30 years ago by Bohandy et al. [46]. LAB operates as follows: First, a glass plate is coated with a layer of metal or oxide that efficiently absorbs laser radiation. Then, a layer of cells suspended in substances such as water, nutrient medium, or gel (bioink) is applied on top. Subsequently, a laser is fired, causing the metal or oxide layer to rapidly heat up and absorb the laser energy. This intense heat generates a vapor bubble within the bioink, which becomes highly pressurized. As the bubble expands, it propels a forceful jet that transports a small droplet bioink onto an acceptor surface [45].

LIFT-based techniques enable precise placement of mammalian cells, especially human cells, with high viability [47, 48]. This breakthrough makes it possible to construct intricate tissues, and paves the path for developing artificial organs [49]. Researchers have found that utilizing LIFT for transferring living cells is rapidly advancing, particularly in the field of biomedicine [50]. However, recently researchers have also proposed the application of LIFT for analyzing microbial cells. This innovative approach offers promising prospects for several valuable outcomes. Firstly, it enables the isolation of novel microorganism species, thereby expanding the knowledge of microbial diversity [47, 51]. Secondly, LIFT-based techniques facilitate the study of the interaction between different microorganisms [12] and their metabolism at the individual cell level [52]. Therefore, utilization of LIFT in the bioprinting of microorganisms holds significant potential for advancing the understanding of microbial systems.

The main advantage of laser-assisted bioprinting is that it has the highest resolution among the different printing principles, which can reach the micrometer level [20]. Furthermore, it is possible to print within a wide range of viscosity (1–300 mPa s^−1^) without the danger of clogging the nozzle, as it is a nozzle-free technique [53, 54]. LAB can print with highly dense bioinks (up to 10^8^ cells per ml) with microscale resolution of a single cell per drop using a laser pulse repetition rate of 5 kHz, with speeds up to 1600 mm s^−1^ [55]. This can be translated to 5000 droplets deposited on the substrate per second [54, 55], which makes LAB the most precise technique among the different bioprinting methods.

One risk of LIFT arises if the energy-absorbing layer consists of harmful substances, as residues of this layer can be transferred during printing [56–58]. However, this disadvantage can be overcome by choosing a non-toxic material to form the absorbing layer [12]. It must be pointed out that using LAB is much more expensive compared to other bioprinting technologies [59].

A comparison of the printing principles extrusion, inkjet, laser-assisted bioprinting and stereolithography can be found in Table 1. It also includes the different biomaterials commonly used for each printing method, which will be described in detail in the following.

**Table 1:** Comparison of the different bioprinting principles

Metric	Extrusion	Inkjet	VAT photo-polymerization	Laser-assisted
Advantages	Versatility	Low cost, modification of 2D inkjet printers	Nozzle free, high resolution, crosslinking larger surface area at the same time	Nozzle free, high resolution
Disadvantages	Mechanical damage at the nozzle, low resolution	Mechanical damage at the nozzle	Not versatile	Metallic residues from energy absorbing layer
Speed	Slow	Fast	Fast	Medium
Resolution, µm	> 100	50	5	1–10
Cell viability, %	40–80	> 85	85	> 90
Viscosity, mPa s^−1^	30 − 6 × 10^7^	3.5–12	No limitation	1–300^1^
Biomaterials	Alginate, gellangum, hyaluronic acid, agarose, PEG	Alginate, PEG	Hyaluronic acid	Collagen
Cost	Low to medium	Low	Low	High
References	[2, 17, 18, 60–65]	[29, 38, 66–69]	[5, 41, 42, 70]	[12, 20, 54, 59, 71, 72]

## Bioink

The ideal composition of the bioink is highly dependent on what microorganisms are being used and what environment should be provided for them [6, 73]. Also, the bioprinting application has an impact on what biomaterials work best for the printing process [6, 74]. The most important materials suitable for each printing method are summarized in Table 1. The biomaterials applied in the bioprinting process are either natural or synthetic polymers or natural biological macromolecules and need to have good physical properties for printability and biocompatibility to provide a stable, non-toxic environment for cell function and -growth [6, 75, 76].

The stability of the 3D bioprinted constructs can be achieved by crosslinking the polymers [77, 78]. Due to the many opportunities to choose for the material, the way of crosslinking and the printing method, there is no universal bioink formula [6, 79, 80]. However, extracellular matrix (ECM) polymers are often used for this purpose [81].

**Fig. 2:**
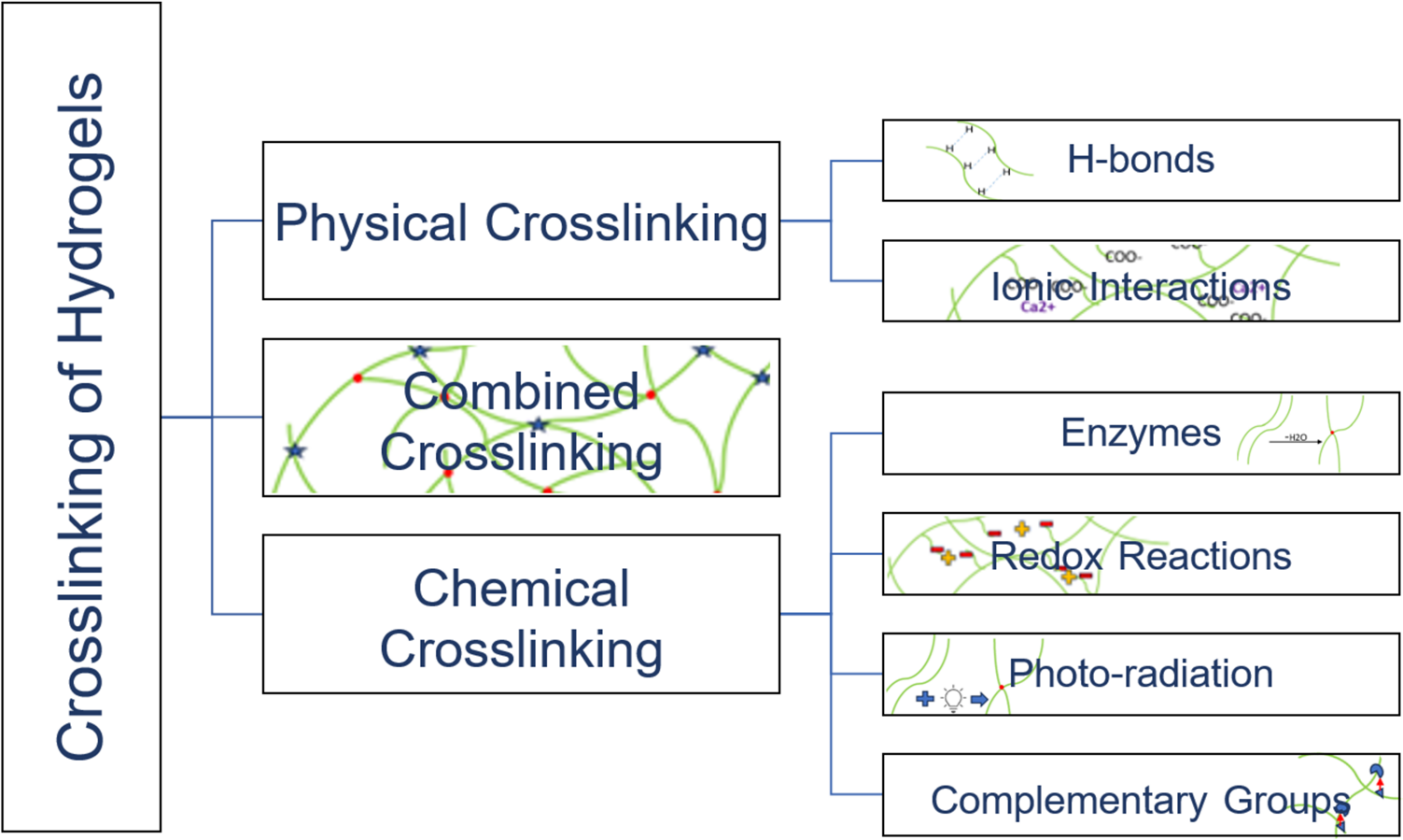
Schematic overview over the most important crosslinking methods, which can be divided into physical crosslinking, chemical crosslinking, and combined crosslinking. The most relevant physical crosslinking methods are via H-bonds and via ionic interactions and the most relevant chemical crosslinking methods are via enzymes, redox reaction, photo-radiation and the reaction of complementary groups

Crosslinking significantly influences the properties of 3D bioprinted constructs, especially the stability and biocompatibility of the environment of the microorganisms [82, 83]. As shown in Fig. 2, crosslinking can be divided into physical and chemical crosslinking, which can also be combined [83, 84]. The most important physical crosslinking strategies include crosslinking via hydrogen bonds or ionic interactions. For the chemical strategies, the crosslinking can be achieved via enzymes, redox reactions, photo-radiation and the reaction of complementary groups [6, 84–89].

The goal for the bioink used in the 3D bioprinting process of microorganisms, is to find a crosslinking method that provides fast gelation and stability of the hydrogel while providing physiological conditions and a non-toxic environment for cell encapsulation [86]. In the following, the crosslinking strategies are explained in detail while elaborating their advantages and disadvantages.

### Physical crosslinking

Gel formation and therefore the stabilization via physical crosslinking can be induced via environmental changes to a specific pH or temperature and result in the formation of reversible intermolecular reactions or H-bonds [77, 78, 85].

Crosslinking of hydrogels via ionic interactions is based on the interaction of two molecules with opposite electrostatic charges [77]. Therefore, positive charged metal ions, like Mg^2+^ or Ca^2+^, interact with the negatively charged acid residues of the polymer that builds the main 3D bioprinted construct, for example alginate [85, 90]. The addition of the metal ions to the polymer solution has a significant impact on the cell viability of the microorganisms and the printability of the bioink [91]. Bath-assisted bioprinting, for example, where the 3D construct is directly printed into a bath with an ionic solution, displays high stability, due to fast gelation. A major drawback of this method is the complex preparation of the crosslinking agent, which needs to be executed very accurately [92]. The major advantage of crosslinking by spraying an aerosol of metal ions on the 3D construct while printing, is the maintenance of high cell viability and reproducibility [92]. The crosslinking agent can also be added before the printing process, where the gelation then is induced via thermal gelation [23].

Hydrogels can be anionic, cationic, and neutrally charged [93]. The backbone of the polymer has naturally ion-groups that can form bonds due to electrostatic interactions when oppositely charged polymers are printed together. This natural way of crosslinking is non-toxic for cells and no additional agents need to be provided and added to the printing process, which also results in a safe environment for microorganisms [94].

This most frequently used method of crosslinking hydrogels has good mechanical properties and biocompatibility but is limited to the electrostatic interactions [78]. The advantages of this method are fast gelation, and mild, physiological reaction conditions, that provide a stable environment for cell encapsulation [84].

A cell-friendly crosslinking method includes non-covalent reactions, like the formation of H-bonds. Song et al. [87] analyzed strategies for high-performance crosslinking of polymers via H-bond crosslinkers and via self-associated H-bonds, which show self-healing properties and high elasticity, and therefore great stability. H-bond crosslinkers are for example small molecules with high amounts of aminogroups, nanoparticles with oxygen-rich surfaces, or polymeric aggregates that have itself high affinity to form many H-bonds. This method has thermostability, self-healing properties and robustness, but a challenge in synthesizing and the current limitation to thermoplastic elastomers. As an example, these hydrogels come to use for self-healing concrete using microorganisms [88].

The advantages of physical crosslinking in general are, that normally no organic crosslinking agents need to be used, and therefore the risk of contaminations and rising toxicity levels due to chemicals can be avoided [83]. Furthermore, the crosslinking process can be achieved under mild, physiological conditions [77]. In the Freeform Reversible Embedding of Suspended Hydrogels (FRESH) printing technique, an example of particular interest within the physical crosslinking methods, a bio-ink is directly printed into a gelatin microparticle carrier bath, undergoes cross-linking, and subsequently incubates at physiological temperature, while this incubation liquefies the gelatin carrier bath, enabling the release of the printed construct [89].

### Chemical crosslinking

Chemical crosslinking is either achieved via crosslinking agents or via naturally formed covalent bonds. The formation of these bonds can be induced by free radical polymerization, enzymatically or by the reaction of complementary groups [78, 91].

The basis of catalytic crosslinking is the linkage of protein-based polymers via enzymes. This method provides mild reaction conditions and, therefore, results in higher cell viability [95]. Wei et al. [96] investigated the swelling behavior of an enzymatically crosslinked hydrogel in relation to a pH-shift. Therefore, they linked poly(γ-glutamic acid) altered with tyramine via horseradish peroxidase in the presence of hydrogen peroxide. They discovered that the pH is corresponsive with the biodegradation rate of the polymer network and the gel is responsive in solutions with pH 2 and pH 7.

Photo crosslinking is a relatively simple and frequently used method and can either be initiated via chain-growth, step-growth or redox reactions [84]. UV-light radiation is the most commonly used photo crosslinking method, thus it can harm microorganisms [78, 89, 97]. To avoid a non-suitable environment, wavelengths of visible light should be used for photo-radiation, in combination with a photo-initiator in low concentrations to provide good cell viability [98, 99]. All light-specific parameters need to be optimized in relation to the materials used, such as intensity, wavelength and exposure time [84]. Wu et al. [100], for example, created a polymeric network of gellan gum, photo-crosslinked with polyethylene glycol diacrylate, to provide a stable hydrogel with healing properties.

Free radicals are produced via photo-radiation, in the chain-growth crosslinking method. These radicals interact with functional groups of the polymers and create an irreversible network between the chains [78, 101]. The three steps of step-growth crosslinking include initiation, propagation, and termination. This method is based on alkyl-sulfide crosslinking, therefore, the functional -SH groups of thiols bind to C–C double bonds, triple bonds, or epoxy-groups. The advantage of this method is very fast gelation within up to 1–3 s, but the stability of these constructs is not very high, due to the oxidation of the disulfide bonds [102].

De Grave et al. [103] compared the step-growth and the chain-growth crosslinking method and discovered that there is an influence on the kinetics and stiffness of the polymeric network. Step-growth showed faster kinetics and greater swelling behavior, while chain-growth showed higher storage modulus.

Crosslinking of hydrogels via the reaction of complementary groups can be achieved by Diels–Alder (DA) reaction. This method provides high stability but is limited to slow gelation rates [86]. The DA-reaction is based on a cyclohexene-building mechanism, with which thermostable, smart and self-healing polymers can be synthesized [104]. Madl et al. [86] discovered a method for chemical crosslinking via DA-reaction that showed an increase in gelation rates and hydrolytic stability that provides good cell encapsulation, compared to other DA-crosslinking methods. Therefore, they paired fulvene, which is an electron-rich cyclic diene, with maleimide dienophiles, to form a stable and improved cell-encapsulating polyethylene-glycol hydrogel.

Redox-based crosslinking is a relatively new method and currently not well established in 3D bioprinting. This strategy involves oxidation reactions, that are induced by light, and therefore form reactive radicals that further bind the polymers to networks with high stability [84, 105]. This method is mostly described in secondary crosslinked polymer networks [97], because of the relatively simple controlling of the crosslinking process, due to redox reactions and the changing of the oxidation state of the ionic crosslinker to a higher or lower level [106].

The advantage of chemical crosslinking is the creation of permanent and strong linkages [78], but radiation or crosslinking agents need to be deliberately considered because of their potential to damage the microorganisms [84].

### Combined crosslinking (secondary crosslinking)

Secondary crosslinking is the combination of two crosslinking methods, to achieve, for example, higher stability or improved cell protection, by overcoming the limitations of one crosslinking strategy [6, 76, 97, 107, 108]. Roh et al. [107] combined covalent crosslinking for self-healing properties, and ionic crosslinking for mechanic stability, to fabricate a polymeric network that has the ability to restore stress-induced breakage of the gel, that emerged during the printing process, without the use of organic crosslinking agents.

Seto et al. [108] discovered that an increased density of cross-linked collagen, in combination with free radical scavengers, protects the microorganisms from damage due to photo-radiation, and therefore displays a radio-protective method of crosslinking with increased stability.

Sun et al. [97] investigated “smart materials” that can respond to environmental changes, like pH or temperature, and therefore designed a hydrogel with a thermo-responsive switch. The hydrogel is based on secondary crosslinking, where the first crosslinking method is via chemical crosslinking of 1-vinyl-3-(carboxyethyl)imidazolium chloride to a poly(N-isopropylacrylamide) network. The second crosslinking is executed via electrostatic interactions of iron ions with the carboxyl groups of the polymer network. The thermo-responsive switch is based on the thermosensitivity of poly(N-isopropylacrylamide), and the crosslinking process is initiated due to heat-induced water loss of the polymer network. Therefore, the density of the construct increases due to shrinkage.

Combined crosslinking methods are promising for bioprinting, because of the possibility of induced crosslinking via environmental changes, a significant improvement in stability, cell protective properties and self-healing features. The main drawbacks are dependent on the crosslinking methods used in the secondary crosslinking process [97, 107, 108]. An overview of the advantages and disadvantages of all crosslinking methods is shown in Table 2.

**Table 2:** Overview of the advantages and the disadvantages of the individual crosslinking methods and the biomaterials that are suitable

Crosslinking method	Biomaterials	Advantages	Disadvantages	References
Physical crosslinking				
H-bonds	Hyaluronic acid	Naturally formed bonds: cell friendly, thermostable, self-healing	Challenging in synthesis, use of organic solvents	[87, 109]
Ionic interactions	Alginate	Fast gelation, high cell viability, reproducibility, thermal induction possible	Complex preparation, limited to electrostatic interactions	[23, 85, 91, 92]
Chemical crosslinking				
Enzymes	Hyaluronic acid	Mild reaction conditions, responsible to environmental changes	Slow crosslinking reaction	[95, 96, 110]
Redox reactions	Hyaluronic acid	High stability, simple controlling	Radical formation	[105, 106]
Complementary groups	PEG, hyaluronic acid, agarose	High hydrolytic stability	Slow gelation rate	[86, 110, 111]
Photo-radiation	Gellan-gum, PEG, hyaluronic acid, collagen	Inducible crosslinking via photo-initiators	UV-light harms microorganisms, optimal conditions highly dependent on materials	[78, 84, 99, 112, 137]
Combined crosslinking	Collagen, PEG	Self-healing properties, improved stability, inducible crosslinking, cell protective properties	Dependent on the individual crosslinking methods used	[97, 107, 108]

## Applications

**Fig. 3:**
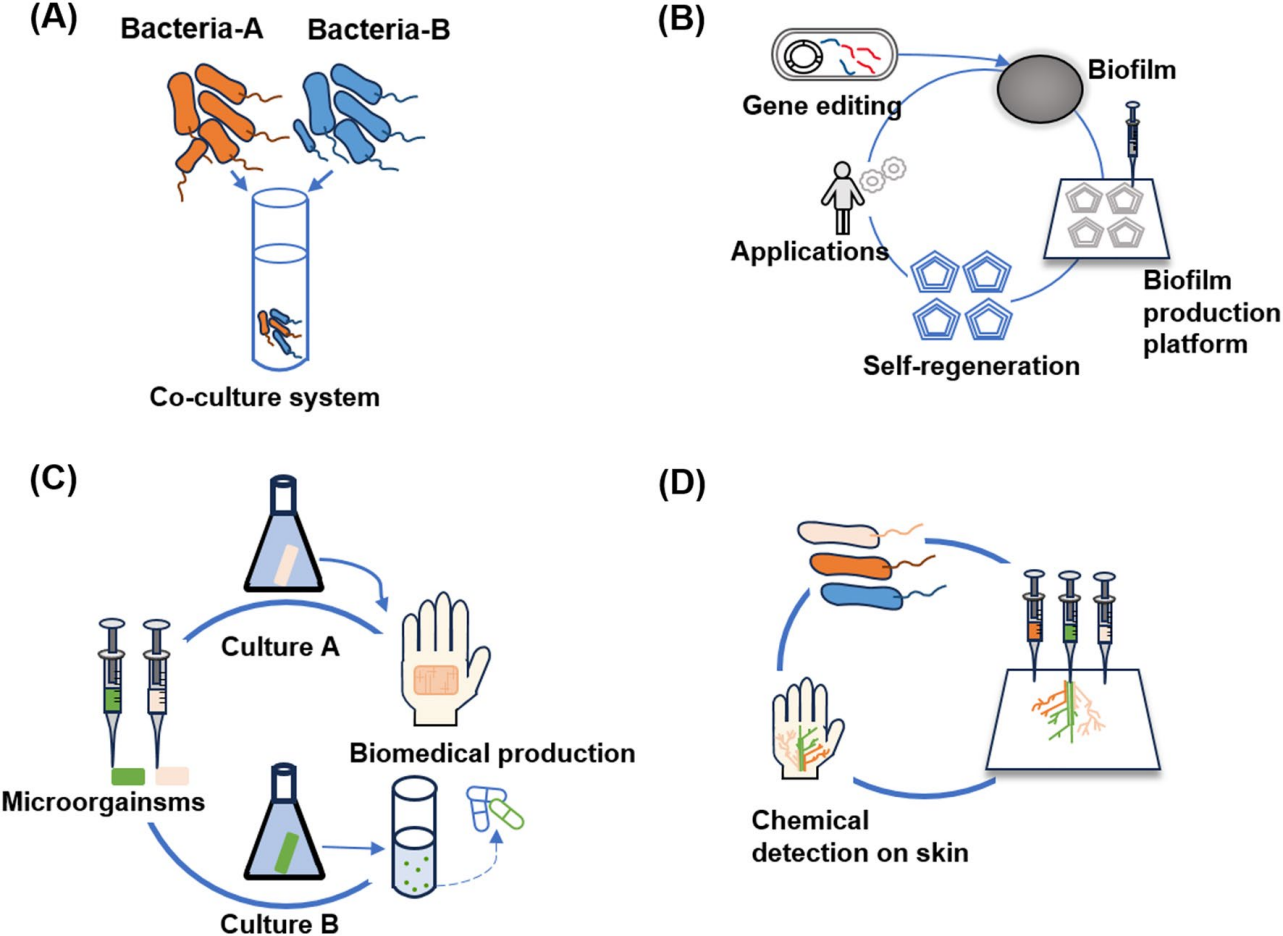
Schematic illustration of the main applications of bacterial bioprinting. **A** Production of bioproducts by 3D printed microorganisms. **B** Construction of artificial biofilms by 3D bioprinting. **C** Production of biomedical materials and medicine. **D** Design of responsive biodevices by 3D bioprinting. Figure illustration on the basis of Liu et al. [6]

With the study of 3D bioprinting technology, the application areas of microorganisms for 3D printing have been explored. Within the scope of this field, scientists combine the physiological characteristics of microorganisms and 3D printing technology to functionalize experimental materials [6]. Bacteria, in particular, can be mixed with biocompatible aqueous solutions that normally contain nutrients and chemicals to form self-supporting hydrogels [6]. In this way, bacteria can be engineered into complex 3D structures with a wide range of potential uses. Figure 3 shows the most important application fields which are the production of bioproducts [113, 114], artificial biofilms [115, 116], biomedicine [117, 118] and responsive devices [119, 120].

### Production of bioproducts

Microorganisms in nature do not work in isolation but rather function in a highly dynamic system of cooperation and competition in which the spatial distribution of microbial communities influences this dynamic. The benefit of 3D bioprinting is the ease with which two or more biological sinks can be quickly printed at predetermined points in space, allowing the development of microbial communities that take into account spatial closeness and the functional complementarity of embedded microbes. Biological sinks in the context of 3D bioprinting refer to designated spatial locations within a printed structure where microorganisms or biological components can be strategically placed. For research in biomolecule production, the construction of spatial combinations of whole-cell biocatalysts can uniquely facilitate the study of microorganisms’ combinations. [6] Microorganisms’ combinations can have a huge impact since co-cultures tend to play an increasingly important role in future production processes [121].

Connell et al. [113] proved the co-culture about sharing of antibiotic resistance within a polymicrobial community containing *Pseudomonas aeruginosa* and *Staphylococcus aureus*, by arranging these two bacterial species’ cells at micrometer length-scales in gelatin using multiphoton lithography. They were inspired by the micro-3D printing technique to build designer ecosystems made for looking at the interactions and integration of various bacterial populations in any 3D structure. In their method, bacteria were added to a warm gelatin manufacturing solution of 37 °C, and by allowing the solution to cool to room temperature, bacteria got suspended at various 3D places throughout the thermally formed gel. After the construction of the co-culture system, they used micro 3D bioprinting to print the co-culture system for further investigations. This 3D printing technique offers the ability to create complex microbial consortia arranged at micrometer resolution in any 3D geometry. A narrowly concentrated pulsed laser beam was scanned in three dimensions to create enclosures around one or more bacteria that meet certain geometric requirements. By combining two or more bacterial species in a single fabrication gel or by progressively bioprinting various cell types using different fabrication gels, communities with multiple populations of segregated bacteria could be created. This study showed that the amount of *P. aeruginosa* needed to surround a *S. aureus* microcolony was sufficient to shield it from antibiotics leaking into the larger community because *P. aeruginosa* is able to produce an enzyme that degrades the antibiotics, allowing *S. aureus* to survive [113].

Building more complicated bacterial catalytic systems has sparked interest due to the site-specific control of bacterial bioprinting on various bacterial distributions. A crucial action was taken by Johnston et al. [122] using a bacterial co-culture system. They created a platform with good mechanical properties that was simple to process and impervious to the biological elements in the media. To create a co-cultured microbial community that was used for the production of high-value-added products, the bioink containing engineered *Saccharomyces cerevisiae* and *E. coli* was printed in a spatially isolated hydrogel. They established a co-culture system using *E. coli* and yeast, wherein red fluorescent (RF) yeast within distinct gel compartments and green fluorescent protein (GFP) bacteria within a combined gel were employed. The two microorganisms were in physical proximity but maintained spatial separation, resulting in a distinct spatial pattern. This spatial patterning enabled to use the benefits of co-culturing, along with enhanced preservation capabilities during freeze drying before fermentation. This co-cultured microbial community performed better than the conventional liquid mixed culture [122].

Lehner et al. [114] first combined alginate and *E. coli* to create a bioink for patterned bioprinting using independently invented extrusion 3D bioprinting. Ionic crosslinking took place by bioprinting on an agar medium containing Ca^2+^ to create a hydrogel. Although significant cell damage occurred during the printing process, activity was recovered within 24 h. Recombinant *E. coli* cells were printed in layers with varying amounts of fluorescent protein expression. Using confocal laser microscopy, good bacterial stratification was seen, and the level of stratification correlated with the level of solidification. This research demonstrated that the two strains were properly constrained in their own areas, laying the groundwork for the development of a bacterial co-culture system [114].

Researchers also considered the production of high-valued bioproducts through the creation of symmetrical 3D printed structures that favor cell growing conditions and bioproduct’s yield. Cui et al. [123] proposed *Streptococcus zooepidemicus* as a bioink model microorganism for the production of bacterial communities in a 3D-printed biofilm bioreactor for the production of hyaluronic acid. The experiment considered the feasibility of the production of macromolecules and how the 3D scaffold orientation impacted the yield. They tested four different layers’ orientations finding that 90 degrees angle layers and an intermediate filament distance produced the highest yield. They used gelatin/methacryloyl bioinks which exhibited good rheological characteristics maintaining an acceptable shape and fidelity [123].

Novel applications of biomolecule production from cell cultures also reach fields such as the construction industry. Reinhardt et al. [124] utilized cyanobacterium *Synechococcus sp.* for its capacity of producing calcium carbonate (CaCO_3_) biomolecules, combined with construction materials like cement. They were able to develop a new type of biocement with self-healing properties. The study describes the creation of living building materials (LBM) through 3D bioprinting techniques. The advantage of using this cyanobacterium is that it can produce a biocement with self-healing properties to be used as a sustainable alternative construction material, reducing the significant impact of the construction industry on global CO_2_ emissions. Calcium carbonate serves as a filling when micro fractures in the cement start to appear. The calcium carbonate present in the cement reacts with water preventing cracks to extend and cause structural weakness. Additionally, the cyanobacterium was able to withstand shear stress and pressure during the extrusion process and remained viable in the immobilized state, making it suitable for use in bioprinted scaffolds. During prolonged cultivation, *Synechococcus sp.* increasingly grew out of the scaffolds with incorporated sand particles into the surrounding cell culture medium. This effect increased with higher sand concentrations. Additionally, alternative support materials to sea sand could be used to further improve the environmental sustainability of the ink, but further research is needed to optimize the bioprinting process and to prevent cell outgrowth [124].

Fungal composites bioink in 3D bioprinting can offer novel topics of research for more sustainable materials and production methods in industries like construction and packaging, replacing traditional materials such as plastics and cements [125]. Fungi poses a high concentration of chitin, an abundant biomolecule with similar properties to cellulose. This creates a final product with similar characteristics to wood or cork. One of the advantages is using very low-cost raw materials from agricultural waste sources such as corn stover and rice straw as candidates to grow fungi biomass, which transforms these raw materials into a network of hyphae [125]. The process consists of 6 stages: (1) the recollection of biomass and fungi from the basidiomycete group, (2) colonization of the material, (3) mixing, (4) 3D bioprinting, (5) secondary colonization and (6) finally drying. The technique preferred by researchers is 3D extrusion in which the most important parameters are extrusion pressure, time of fungi colonization for the hardness between layers and mixing ratio of the bioink. The mixture of the primary colonized fungi-biomass composite is composed of water and wheat flour [126].

### Artificial biofilm

**Fig. 4:**
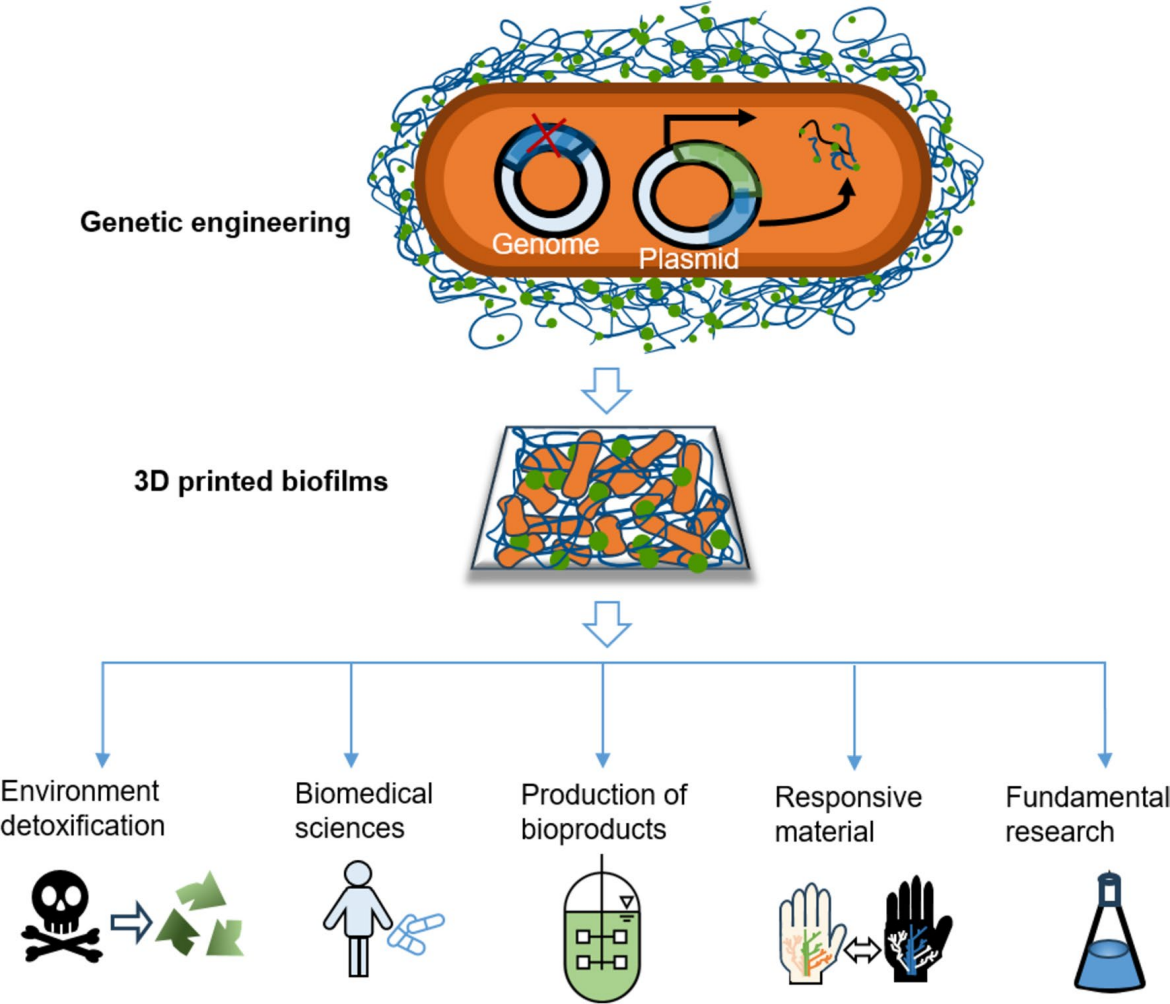
Possible applications for 3D bioprinting of synthetic biofilms: Environmental detoxification, biomedical applications, production of bioproducts, manufacturing of responsive materials and fundamental research. Figure illustration on the basis of Balasubramanian et al. [115]

Due to their adaptability and diverse metabolic activities, bacteria can flourish in almost any ecological niche [127]. Because this metabolic diversity is more abundant than in any other type of organism, bacteria produce physical material in the form of biofilms that ensure survival even in harsh environments [128, 129], such as changes in temperature, pH, and others. Biofilms adjust their mechanical properties under pressure to match the conditions imposed by the surrounding environment with a wide range of biopolymers. Biofilms also could provide a stable structure and suitable environment [130, 131]. During the growth of biofilms, bacteria can also form and degrade a large number of compounds and, in addition, bacteria are able to form calcium carbonate [132], magnetite [133] and biopolymers [134]. Figure 4 shows an overview of applications for 3D bioprinting of artificial biofilms. After engineering the microorganisms’ genetics, they could be printed through the 3D bioprinting technology into artificial biofilms. This offers a wide range of functional properties, providing potential for a wide variety of applications, including the most important applications such as environmental detoxification [135, 136], biomedical production [118, 138, 139], material production [136, 140, 141], responsive materials [119, 120], and fundamental research [138].

Lehner et al. [114] showed how to manufacture germs using basic alginate chemistry with transformed commercial 3D printers or construction toys [114, 132, 137]. Their printing tools used a modified commercially available extrusion 3D printer. The extruded material was a custom bioink that mixed the living bacteria with dissolved alginate. It would enable suspended bacteria and chemical substrates for making materials to pass through the print head and quickly solidify into a stable pattern when it comes into touch with the printing surface. With the aid of simple chemistry and user-friendly technology, they were able to print high-resolution three-dimensional samples that were reproducible [114].

Schmieden et al. [116] combined biofilm-forming bacteria with 3D bioprinting, which resulted in the creation of repeatable, standardized biofilms for scientific studies. They showed a new technique for 3D bioprinting materials made from modified *E. coli* cells that were inspired by biofilms. The bioink was engineered to induce cells expressing the CsgA protein after printing. The CsgA protein functions as a major building block for bacterial curli fibers, contributing to biofilm formation, surface adhesion, and protection of cells within the biofilm structure. A synthetic biofilm was formed that protected cells from being rubbed off by substances that dissolve the gel. The authors created a prototype of a cheap 3D printer for bacteria using K’NEX (K’Nex Industries, Inc, Hatfield, Pennsylvania) components that could print bacteria in layered, stable 3D structures [116].

Since artificial biofilms could be applied in many fields, there are lots of applications that could make the contribution to people’s life. For medical and food production, the best high-water habitat is established for bacterial survival, nutritional inflow, and waste dispersion by encapsulating the bacteria in hydrogels [118]. Bacteria produce hydrogels on their own in the form of barrier biofilms with a variety of mechanical properties. For example, using amyloid fibers, *Bacillus subtilis* creates biofilms at the water–air interface that have rather robust mechanical qualities and are cohesive, making them ideal for use as wound patches. *Bacillus subtilis* bacterium senses signals from *S. aureus* and responds by releasing antibiotics against *S. aureus* [118, 138, 139]. At the water–air interface, other microbes, such *Acetobacter xylinum*, also known as *Gluconacetobacter xylinus*, are able to produce nanocellulose hydrogels with astounding tensile strength [118, 142, 143].

For material production, *Clostridium acetobutylicum* is a well-researched bacterium with a lengthy industrial history that has been suggested as a potential substitute for the production of biofuels [141]. Schmeckebier et al. [144] successfully used *C. acetobutylicum* in artificial biofilms in a laboratory unsaturated flow reactor to produce alternative biofuels like butanol and hydrogen [144, 145]. Napoli et al. [141] used immobilized *C. acetobotylicum* on Tygon (Saint-Gobain Corporation, Courbevoie, France) as a carrier in a continuous packed bed reactor (PBR). The reactor was employed for the production of butanol to demonstrate the potential for sustainable bioprocessing using immobilized cells in a continuous reactor configuration [141].

However, with immobilized cells in bioproduction, there are drawbacks to be taken into account, such as limiting mass transfer in the biofilm and managing biofilm growth [144]. The use of 3D-printed engineered biofilms are promising for environmental purification processes, such as bioremediation, heavy metal and rare earth element extraction, organic carbon removal, and wastewater treatment facilities [118, 146]. Patterned-designed biofilms operate as sinks able to absorb and degrade pollutants by combining the enhanced metabolic potential and particular catabolic features of active bacteria with the increased surface area and chemical flexibility of biofilm matrices [115].

For fundamental research, the unidentified interactions between various bacterial biofilms or between bacterial biofilms and the eukaryotic hosts which they dwell can be discovered using 3D-printed artificial biofilms. The bioink contains various bacteria, and it can be printed in the nearby sharing interface or on existing, tested 3D-printed biofilms. After a sufficient exposure time, imaging tools and histology methods can then be used to understand the communication and social behavior of bacteria and their hosts [115].

The management of viral diseases or the development of new antibiofilm medications might benefit from these foundational studies. As an example, *Bacillus subtilis* was employed because of its ability to produce strong biofilms, its ability to secrete proteins that can alter cell activity, and its genetic tractability [147]. Because of its genetic tractability, *Bacillus subtilis* has also demonstrated efficacious utilization in the fabrication of synthetic biofilms and engineered materials [115].

### Biomedical applications

Bacteria can produce and breakdown a wide range of compounds that are frequently used in the production of chemicals, biopolymers, enzymes, and proteins relevant to the medical sectors. [117]. Compared to free culture, microbial immobilization has a number of advantages, including high production efficiency, resistance to potentially harmful environmental chemicals, and ongoing usage and recycling. 3D bioprinting technology, as a new immobilization technology, unquestionably has greater potential in the production of medical materials because it can produce more customized products based on improved microbial living environments in accordance with preferences and practical application scenarios. This results in additional application domains for immobilization technologies in this field [6].

Szarlej et al. [148] used the efficacy of a 3D composite polyurethane-polylactide (PUR/PLA) flexible filaments scaffold as a potential wound dressing by assessing cell growth and improving antimicrobial effects for skin regeneration and bone graft using *Staphylococcus aureus* bacteria as a model microorganism. *S. aureus* is a common bacterium found on human skin that can be easily cultured in the laboratory. Researchers combined the extrusion of PLA and thermoplastic polyurethanes (TPUs) with the antibacterial activity of amikacin to test the release profile and its effectiveness on bacterial cultures. Results showed that amikacin survived the extrusion process and reduced bacterial growth over PLA and TPUs 3D printed structures [148].

3D cellulosic structures using the cellulose-producing bacteria *Acetobacter xylinum* were shown to be very helpful in the medical field due to the biocompatibility of bacterial cellulose [118, 149]. Bacterial cellulose has been produced in situ as wound dressings [118, 148], prospective blood arteries [142, 150], and surface-patterned implants [149].

**Fig. 5:**
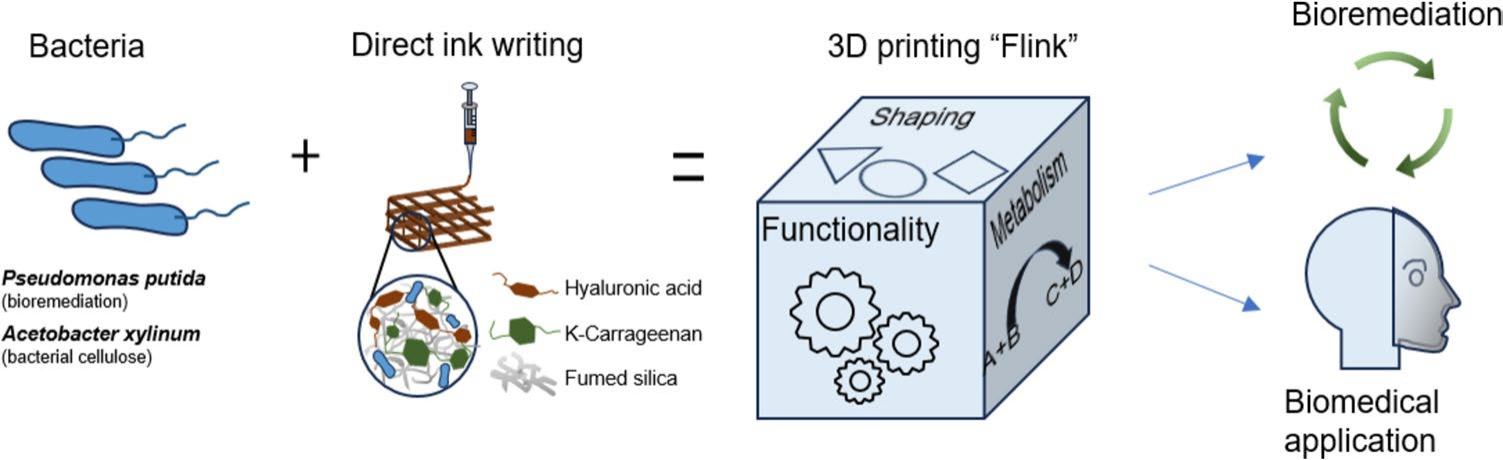
Schematics of the 3D bacteria-printing platform for the creation of functional living materials (flink). The incorporation of particular bacterial strains results in the development of a dynamic and responsive hydrogel, representing a new class of material termed “flink”. Figure illustration on the basis of Schaffner et al. [118]

Schaffner et al. [118] developed a novel type of composite bioink called “functional living ink (flink)”, which uses hyaluronic acid, k-carrageenan, and fumed silica to mix in a certain proportion to maintain good viscoelasticity while having the ability of shear thinning for extrusion-based printing. *Pseudomonas putida* and *A. xylum* were used as model organisms for the creation of functional and complex hydrogels. *P. putida* is a well-studied bacterium that is known to have a high metabolic versatility and can grow on a wide range of substrates such as phenols [118]. Figure 5 depicts the workflow of “flink,” which displays its bioremediation and biomedical applications.

To make hydrogels with sufficient mechanical strength, hyaluronic acid (HA) can be substituted with glycidyl methacrylate HA (GMHA) [6]. The substitution of HA with GMHA does not induce notable changes in viscosity but enables the hydrogel to undergo UV-cross-linking at a low exposure dose and harmless wavelengths (365 nm for 60 s at 90 mW). Environmentally hazardous phenols were broken down using *P. putida* immobilized in a 3D printed “flink”-GMHA grid [118].

Schaffner et al. [118] also presented a methodological innovation utilizing 3D bioprinting techniques to fabricate functional materials derived from bacteria, leveraging their diverse natural metabolism. This approach seamlessly integrates the intrinsic metabolic capabilities of bacteria with the design flexibility offered by additive manufacturing processes. To realize this amalgamation, they encapsulated *Pseudomonas putida* and *Acetobacter xylinum,* among others, within a biocompatible and functionalized 3D printing ink called functional living ink (flink), enabling the production of two distinct categories of living materials. These materials exhibited efficient degradation of environmental pollutants and the synthesis of medically pertinent bacterial cellulose. The versatility of this bacterial printing platform enabled the construction of complex materials with precise spatial configurations, compositions, and properties. Thus, they were able to print a face mask made of “flink” for a doll, which perfectly fit the doll’s facial contours. This opened up further possibilities for biomedical applications [118].

### Responsive devices

Biocompatible aqueous solutions that typically include nutrients and chemicals can be combined with bacteria to create self-supporting hydrogels. This enables the production of complicated 3D structures with a wide range of possible applications [151].

Liu et al. [119] presented living sensors by modifying *E. coli* and patterning hydrogels. Through genetic modification of *E. coli* within the hydrogel structure, specifically engineered to sense chemical inducers present in human skin (N-acyl homoserine lactone, isopropyl-D-1-thiogalactopyranoside, and rhamnose), the researchers facilitated the 3D printing of wearable materials. The development of ingestible or implanted sensors have the potential to modulate the gut microbiota and address micro-mediated diseases such as obesity and diabetes, which would be an intriguing application of this technology [119].

Mcbee et al. [120] described a live tattoo out of regenerative fungal–bacterial biocomposite structures for chemical detection, where a small layer of elastomer was used to print the tattoo as a tree-like design, which was then applied to the human skin. The tattoo was manufactured using 3D printing. They utilized food dyes to enhance the visualization of the hydrogel pattern. The different cell types encapsulated were differentiated by hydrogels in distinct colours. Additionally, the tattoo-responsive devices were coated with different activated small biological molecules like Rham, IPTG, or AHL. Due to the fact that the living sensors contained different molecules in the tattoo, they could generate fluorescence in response to certain substances. For instance, alterations in the skin’s state through compression, stretching, or twisting demonstrated discernible responses in the optical properties of the living tattoo, manifesting as variations in lightness or darkness. These mechanisms may assist individuals in perceiving distinct conditions, as the responsive devices manifest diverse visual expressions [120].

Table 3 gives a summary of the most important applications for 3D bioprinting in different areas covered in this review.

**Table 3:** Overview of the most important applications, the microorganisms and the immobilization techniques used

Application	Objective	Microorganisms	Immobilization technique	References
Production of bioproducts	Production with co-cultures	*Pseudomonas aeruginosa* and *Staphylococcus aureus*	Multiphoton lithography	[113]
		*Saccharomyces cerevisiae* and *E. coli*	Direct-write extrusion printing	[122]
	Basic research	*E. coli*	Extrusion based bioprinting	[114]
		Undefined biomass-fungi mixture	Extrusion based bioprinting	[126]
	Hyaluronic acid production	*Streptococcus zooepidemicus*	Photocuring	[123]
	Calcium carbonate production	*Synechococcus sp.*	Extrusion based bioprinting	[124]
Artificial biofilm	Basic research	*E. coli*	Extrusion based bioprinting	[114, 116]
		*E. coli, Pseudomonas fluorescens and Bacillus subtilis*	Self-growing biofilms	[138]
	Development of novel printing technique	*Pseudomonas putida* and* Acetobacter xylinum*	Novel printing technique	[118]
		*E. coli*	Novel printing technique	[115]
	Development of a novel reactor	*Clostridium acetobutylicum*	Growing on carrier material	[141]
Biomedical application	Production of antibacterial wound dressings	*E. coli*, *P. fluorescens, S. aureus* and *S. epidermidis*	Fused filament fabrication	[148]
	Basic research	*A. xylinum*	Cell adhesion	[142]
Responsive devices	Development of wearble materials	*E. coli*	Direct writing and UV curing	[119]
	Basic research	Different fungi and bacteria in composites	Different types	[120]

## Conclusion and future perspectives

This review gives an overview of the state-of-the-art of 3D bioprinting-methods and applications, as well as microorganisms that are used for bioprinting, and about crosslinking methods for polymeric networks. The combination of the printing technology used, the choice of the microorganisms and the bioinks, depends highly on the application and the aimed product. Bioinks need to have good printability, have to be suitable for the chosen printing device, and have to display mechanical integrity, stability, and biocompatibility. The way of crosslinking of the polymeric network of the bioink is dependent on the microorganism and the printing method, and what physical and physiological properties are aspired and needed, to provide a stable environment for cell encapsulation. The most important encapsulation characteristics are elasticity, stability, and physiological conditions.

Applications of 3D bioprinting rapidly increased during the last years and are being constantly improved for the production of bioproducts, responsive devices, biomedicine, and many others. For example, tissue repair and regeneration, as well as biosensors designed to detect early signs of diseases or infections, showcase this potential. In the future, we believe that with the development of microorganisms’ 3D printing techniques, there will be more applications waiting for people to discover. For example, solar-driven air recycling systems may be achieved by bioprinting photoautotrophic microorganisms (microalgae, cyanobacteria) into transparent facade elements of buildings to exchange CO_2_ for O_2_ by passing consumed CO_2_-enriched air through these elements to produce O_2_-enriched air for recycling. The variety of microorganisms available for bioprinting also promotes future development in the production of fine chemicals in bioreactors because the diverse array of microorganisms available for 3D bioprinting facilitates precise control over biochemical cascade production, allowing for specialized syntheses, activation of synergistic effects by tailored microbial consortia, enabling customizable bioreactor designs, and resource-efficient scalability. Another interesting perspective is the inclusion of machine learning techniques into the bioprinting of microorganisms. They could help for example with design optimization, printing process improvement, quality control or bioprinting customization.

In conclusion, 3D bioprinting of microorganisms is an emerging technology with wide applications and promising prospects. Although it is still in the exploratory stage, its potential value has attracted extensive attention in the fields of medical treatment, biomanufacturing, and environmental protection. With further research and development, 3D printing of microorganisms is expected to bring significant advances to industry and society.

## Data Availability

Not applicable.

## Abbreviations

3D: Three dimensional
DA: Diels–Alder
GelMA: Gelatin methacryloyl
GFP: Green fluorescent protein
H-bonds: Hydrogen bonds
LAB: Laser-assisted bioprinting
LIFT: Laser induced forward transfer
PEG: Polyethylen glycol
PUR/PLA: Polyurethane-polylactide
RF: Red fluorescent
SLA: Stereolithography
SLAM: Stereolithographic apparatus for microbial bioprinting
TPUs: Thermo plastic polyurethanes
